# Supplementary material for: The impact of cultural tourism experience on cultural identity: A case study of Mazu culture
Source: PLoS One. 2026 Apr 2;21(4):e0346153. doi: 10.1371/journal.pone.0346153 (PMC13046174; doi:10.1371/journal.pone.0346153)
Supplement: S1 Appendix — This table contains the complete wording of all survey items, along with their descriptive statistics (means and standard deviations), standardized factor loadings, and standard errors. (DOCX) [file pone.0346153.s001.docx]

**Supporting information**

S1 Appendix. Measurement item details for the study. This table contains the complete wording of all survey items, along with their descriptive statistics (means and standard deviations), standardized factor loadings, and standard errors.

| Construct | Code | Item | Mean | SD | Std. Loading | S.E. |
| --- | --- | --- | --- | --- | --- | --- |
| Entertainment Experience | Ent1 | The Mazu cultural activities (e.g., festivals) were enjoyable. | 3.79 | 0.773 | 0.749 | 一 |
|  | Ent2 | I found the recreational activities at the Mazu sites to be fun. | 3.64 | 0.785 | 0.799 | 0.065 |
|  | Ent3 | Participating in these activities made me feel excited. | 3.68 | 0.798 | 0.739 | 0.064 |
| Educational Experience | Edu1 | The visit helped me acquire knowledge about Mazu's history. | 3.71 | 0.712 | 0.780 | 一 |
|  | Edu2 | I gained a deeper understanding of Mazu beliefs and customs. | 3.75 | 0.723 | 0.747 | 0.056 |
|  | Edu3 | The experience enhanced my knowledge of Mazu culture. | 3.77 | 0.770 | 0.813 | 0.061 |
| Escapist Experience | Esc1 | The Mazu cultural environment allowed me to escape from my daily routine. | 3.59 | 0.881 | 0.833 | 一 |
|  | Esc2 | I felt completely immersed in the spiritual atmosphere of Meizhou Island. | 3.65 | 0.890 | 0.748 | 0.051 |
|  | Esc3 | I experienced a sense of psychological detachment and spiritual transcendence here. | 3.62 | 0.896 | 0.773 | 0.051 |
| Esthetic Experience | Est1 | I admired the sensory and artistic appeal of the Mazu temples and landscapes. | 3.57 | 0.707 | 0.766 | 一 |
|  | Est2 | The overall ambiance and esthetic of the Mazu sites was pleasing. | 3.59 | 0.677 | 0.710 | 0.062 |
|  | Est3 | I appreciated the architectural beauty and natural surroundings. | 3.60 | 0.737 | 0.664 | 0.066 |
| Emotional Resonance | ER1 | My experience with Mazu culture struck a deep emotional chord with me. | 3.61 | 0.856 | 0.634 | 一 |
|  | ER2 | The values of Mazu culture resonated with my personal values. | 3.51 | 0.837 | 0.734 | 0.084 |
|  | ER3 | This cultural experience left a lasting emotional impression on me. | 3.52 | 0.865 | 0.791 | 0.091 |
| Cultural Identity | CI1 | I feel a sense of belonging to the Mazu cultural community. | 3.62 | 0.836 | 0.767 | 一 |
|  | CI2 | I feel proud of the Mazu culture. | 3.65 | 0.852 | 0.815 | 0.058 |
|  | CI3 | I identify with the values of Mazu culture. | 3.64 | 0.887 | 0.784 | 0.06 |
| Place Identity | PI1 | Meizhou Island, as the birthplace of Mazu, holds special symbolic meaning for me. | 4.34 | 0.644 | 0.708 | 一 |
|  | PI2 | I feel a strong emotional attachment to Meizhou Island. | 4.36 | 0.682 | 0.749 | 0.072 |
|  | PI3 | I feel that Meizhou Island is a part of my identity. | 4.31 | 0.700 | 0.742 | 0.074 |
