# Supplementary material for: The impact of cultural tourism experience on cultural identity: A case study of Mazu culture
Source: PLoS One. 2026 Apr 2;21(4):e0346153. doi: 10.1371/journal.pone.0346153 (PMC13046174; doi:10.1371/journal.pone.0346153)
Supplement: Attachment — Submitted filename: Response to Reviewers.docx [file pone.0346153.s005.docx]

Response to Reviewers

Dear Dr. Zhu and Reviewers,

Re: Manuscript ID PONE-D-25-53833

Title: The Impact of Cultural Tourism Experience on Cultural Identity: A Case Study of Mazu Culture

Thank you for the opportunity to revise and resubmit our manuscript. We sincerely appreciate the editors and reviewers for their time, insightful comments, and constructive suggestions, which have significantly strengthened our paper.

We have carefully considered all comments and have revised the manuscript accordingly. Below, we provide a point-by-point response to each comment. All changes in the manuscript have been highlighted in the ‘Revised Manuscript with Track Changes’ file for your easy reference.

**Response to Comments from Reviewer #1:**

**Reviewer #1 Comment 1:** Introduction: The objectives should have been defined more clearly.

**Response:** We thank the reviewer for this helpful suggestion. To enhance clarity, we have revised the Introduction to explicitly and concisely state the study’s objectives in a dedicated paragraph. The new text on page 5, now reads:

Consequently, this study aims to achieve three specific objectives: **(1) To examine how the four dimensions of Mazu cultural tourism 4E experiences differentially influence tourists' emotional resonance. (2) To test the sequential mediating roles of emotional resonance and cultural identity in the relationship between 4E experiences and place identity. (3) To advance the S-O-R paradigm by unpacking the internal psychological sequence "black box" of identity formation in folk-religious cultural tourism.**

We believe this revision provides a clearer roadmap for the reader and more directly aligns with the research questions and hypotheses that follow.

**Reviewer #1 Comment 2:** The literature review is extensive and lacks a clear framework that specifically addresses cultural tourism experiences. While the experience economy and cultural tourism are tightly interconnected, they stem from distinct theoretical backgrounds. This distinction should be reflected in your review, but it wasn't. Additionally, much of the argumentation here focuses on justifications. For example: "Remains limited, and the scholarly understanding of its unique dimensions and linkages to psychological outcomes is underdeveloped [12]."

"Despite this understanding, a significant research gap persists. Many studies treat emotion generically, failing to delineate the specific mediating role of states like resonance in translating experiences into enduring identity formation [12,15,39]."

"Directly addressing this gap and ambiguity, and in alignment with the S-O-R framework, our study conceptualizes emotional resonance as the critical internal organismic state (O)."

This is repetitive and already mentioned in the introduction. The literature review should focus on presenting the state of the art regarding theoretical frameworks and theories, not merely pointing out research gaps and continuously justifying how your study addresses them. If you had integrated your hypotheses into this section (rather than leaving them to a separate "Hypothesis Development" section), it would make more sense. By the way, it should be hypotheses (plural), not hypothesis (singular).

**Response:** We have thoroughly revised the “Literature review and hypotheses development” section as follows:

**Providing a Clear Theoretical Framework:** We have restructured the entire literature review around the overarching **Stimulus-Organism-Response (S-O-R) framework.** Within this paradigm, we explicitly introduce and distinguish the roles of **Experience Economy Theory** (providing the universal 4E typology) and its application within the specific domain of **cultural tourism theory.** This clarifies the theoretical grounding of our core construct, Mazu cultural tourism experience.

**Focusing on State-of-the-Art Synthesis:** Following the reviewer’s advice, we have removed all repetitive statements that merely pointed out research gaps or justified our study’s position, which were indeed redundant with the Introduction. The revised section now focuses squarely on synthesizing relevant theories (e.g., Resonance Theory, Broaden-and-Build Theory, Social Identity Theory, Place Attachment Theory) and prior empirical findings to build a logical foundation for our model.

**Integrating Hypotheses Development:** We have merged the standalone hypothesis development into the literature review. The hypotheses (H1–H19) are now logically derived and presented at the end of their respective theoretical subsections. This integration ensures a seamless flow from theoretical discussion to specific, testable propositions, enhancing the narrative coherence.

**Correcting Terminology:** All instances of “hypothesis” have been corrected to the plural form “hypotheses” throughout the text.

We believe these comprehensive revisions have transformed the literature review into a more focused, theory-driven, and logically flowing section that effectively sets the stage for our empirical study.

**Reviewer #1 Comment 3:** Methods: The section on measurement items should be renamed to Data Design. There's no need to repeat the survey items you’ve already listed in Table 1. Instead, it would be more effective to explain the type of questionnaire used and justify why you chose it.

**Response:** We have revised the “Methods” section accordingly. The subsection previously titled “Measurement items” has been renamed to **“Data Design”.** Following the reviewer’s suggestion, we have removed the repetition of survey items within the text and instead focused on explaining the design of the structured questionnaire. We now detail the use of a five-point Likert scale, the adaptation of measurement scales from established literature to fit the Mazu cultural context, and the justification for selecting these specific scales based on their psychometric properties and applicability in tourism studies. Readers are directed to Table 1 for the complete item wording. These changes are reflected in the “Data Design” subsection of the revised manuscript.

**Reviewer #1 Comment 4:** Data Collection:

The information about Meizhou should be placed in its own section, such as "3.1. Setting". While this isn't mandatory, since it has been increasingly common recently in articles.

The Data Collection section should focus on the methodology of how you gathered your data.

Be clearer about the “tourists.” Are you referring to all tourists? Does this include both nationals and foreigners? Are participants only those over 18? I also recommend moving Table 2 to the Results section, as it pertains to the findings, not the methodology. Lastly, there is no mention of the nationality of the tourists. This would be an important detail to include.

Also not information was given to how the questionnaires were applied, where, by whom and why?

**Response:** We have thoroughly restructured the “Methods” section to address these points:

A dedicated subsection titled **“Study Setting: Meizhou Island”** has been created to provide the contextual background separately.

The **“Data Collection and Sample”** section now focuses squarely on methodology. Under the subheading **“Participants and Procedure”,** we clearly specify that the study targeted **adult (aged 18+) domestic tourists,** detail the data collection period (April-August 2025), and describe how the questionnaires were administered face-to-face by trained assistants at high-traffic locations on the island.

As suggested, **Table 2 (Demographic Profile)** has been moved from the Methods to the **“Results”** section under the heading “Sample Demographics”.

The rationale for targeting domestic tourists (as the primary visitor group for this Chinese folk-religious heritage site) and using a convenience sampling approach to obtain a diverse sample is now explicitly stated in the “Participants and Procedure” subsection.

These revisions ensure clarity regarding the sample, transparency in data collection procedures, and a logical separation between methodological description and research findings.

**Reviewer #1 Comment 5:** Results: I noticed that you didn’t include the HTMT (Heterotrait-Monotrait Ratio of correlations), which would be valuable. While the Fornell-Larcker criterion is traditionally used for SEM discriminant validity testing, it’s been considered less robust for complex models for some time now. The HTMT method is a more precise and powerful measure because it can detect discriminant validity issues in models with highly correlated constructs.

**Response:** We agree with the reviewer on the value of the HTMT ratio for providing robust evidence of discriminant validity. Following this suggestion, we have now conducted and reported the HTMT analysis. The results are presented in a new **Table 5 (HTMT matrix) and Table 6 (HTMT inference with 95% bias-corrected bootstrap confidence intervals).** All HTMT values range from 0.401 to 0.670, well below the conservative threshold of 0.85, and none of the confidence intervals include 1. This analysis, along with its interpretation, has been integrated into the “Reliability and validity testing of scales” section, providing strong support for the discriminant validity of our constructs.

**Reviewer #1 Comment 6:** Discussion:

The discussion section is short and lacks depth. I have significant concerns with your claims, particularly given that you used convenience sampling.

The tone of the discussion reads more like a statement, such as: “Our results move beyond the established...” or “This refines the Stimulus-Organism-Response (S-O-R) framework...”. I would argue that your real discussion is in the practical implications section. But, these are two distinct sections.

It’s highly recommended to move the limitations and future research sections into the Conclusion.

**Response:** We have substantially revised the manuscript in response to these important comments:

**Depth and Length of Discussion:** The “Discussion” section has been greatly expanded and enriched. We now provide deeper theoretical interpretation of key findings, including dedicated subsections on the centrality of the escapist experience, the indirect role of educational experience in folk-religious contexts, and the proposal of a refined sequential S-O-R model. This moves beyond mere restatement of results to offer meaningful theoretical integration and explanation.

**Tone:** We have moderated the tone throughout the discussion to focus on interpreting findings within the context of existing literature and theoretical frameworks, rather than making declarative statements.

**Section Restructuring:** Following the recommendation, the “Limitations and Future Research” section has been moved out of the Discussion and now stands as a separate section following the “Conclusion”.

**Acknowledgment of Convenience Sampling:** The limitation regarding the use of a convenience sample is explicitly acknowledged as the first point in the “Limitations and Future Research” section, which addresses the reviewer’s specific concern.

These revisions ensure the Discussion provides the necessary depth and critical analysis, while the manuscript structure aligns with conventional academic formatting.

**Reviewer #1 Comment 7:** Conclusion: Once again, I have serious concerns with your claims, especially given the use of convenience sampling, which limits the generalizability of your results.

**Response:** We have revised the manuscript to directly address these concerns.

The “Conclusion” section has been refined to ensure that our claims are presented as contributions within the context of this study and its theoretical advancements, rather than as overstated generalizations. The language now more carefully summarizes the empirical support found for our model and its implications.

More importantly, the specific limitation regarding convenience sampling and its impact on generalizability is now explicitly acknowledged as the primary point in the dedicated “Limitations and Future Research” section, which follows the Conclusion. This structural change ensures that the boundaries of our findings and the need for future validation are clearly communicated to the reader, directly addressing the reviewer’s valid concern about over-claiming.

**Reviewer #2 Comment 1:** 1- Discriminant validity:

Please report HTMT ratios (with confidence intervals). Fornell–Larcker alone is not sufficient under current SEM standards.

**Response:** As suggested, we have added the Heterotrait-Monotrait (HTMT) ratio analysis to assess discriminant validity. The HTMT matrix (Table 5) and bootstrap confidence intervals (Table 6) are now reported in the “Reliability and validity testing of scales” section. All HTMT values are well below the 0.85 threshold, providing strong evidence for discriminant validity.

**Reviewer #2 Comment 2:** Common Method Bias (CMB): Because all data were collected via a single-source survey, additional diagnostics are necessary. Please include at least one modern remedy (marker variable, CLF test, or latent method factor).

**Response:** We have addressed this concern by incorporating additional diagnostics for common method bias as recommended. In the “Reliability and validity testing of scales” section, we now report the results of two complementary assessments: **(1) Harman’s single-factor test, which indicated that a single factor accounted for 36.1% of the variance (<50%); and (2) a marker variable test, which showed no significant improvement in model fit when the marker variable was included (Δχ²(1) = 0.087, p > 0.05).** Based on these results, we conclude that common method bias is not a major concern for this study.

**Reviewer #2 Comment 3:** 3- Measurement model transparency:

Include full item wording, standardized loadings with SEs, item means/SDs, and VIF values in an appendix. These are essential for evaluating construct validity.

**Response:** To enhance transparency, we have added a comprehensive S1 Appendix. This appendix now provides the full wording of all measurement items, along with their corresponding means, standard deviations, standardized factor loadings, and standard errors, as requested.

Regarding VIF values, they are typically used to assess multicollinearity among observed predictor variables in regression analysis. In our covariance-based SEM analysis, the assessment of discriminant validity and relationships among latent constructs is more conventionally presented through the correlation matrix (Table 4) and the confirmatory factor analysis results (Table 3, S1 Appendix). We believe the information now provided in the S1 Appendix, combined with the existing tables, offers a complete and standard view of the measurement model for evaluation.

**Reviewer #2 Comment 4:** 4- Model fit:

Please report SRMR and briefly justify the fit thresholds used.

**Response:** We have added the SRMR (Standardized Root Mean Square Residual) to the model fit reporting in both the measurement model and structural model (Table 7) sections. Furthermore, we have now explicitly cited the benchmark thresholds used for model fit evaluation (e.g., SRMR & RMSEA < 0.08; IFI/TLI/CFI > 0.90) with reference to Hu and Bentler (1999), as suggested.

**Reviewer #2 Comment 5:** 5- Alternative model comparison:

To justify the sequential mediation, compare your model with at least one plausible alternative.

**Response:** We have conducted the suggested alternative model comparison. In the new “Structural equation modeling testing results” section (and Table 7), we compare our hypothesized sequential model (Model 1: 4E → ER → CI → PI) against two theoretically-derived alternatives: a model with emotional resonance as the sole mediator (Model 2: 4E → ER → PI) and a model with cultural identity as the sole mediator (Model 3: 4E → CI → PI).

The results provide strong support for our proposed model: (1) It demonstrates the highest explanatory power for place identity (R² = .651). (2) While its fit is statistically equivalent to the more parsimonious Model 2, it provides a significantly better fit than Model 3, underscoring the indispensable role of emotional resonance as the initial mediator in the chain. This comparison empirically justifies our proposed sequential mediation pathway.

**Reviewer #2 Comment 6:** 6- Data availability:

PLOS ONE requires publicly available raw data and analysis files. Please deposit the dataset, syntax files, and a README in a public repository.

**Response:** We have fully complied with PLOS ONE's data availability policy. A **“Data Availability Statement”** has been added to the end of the manuscript. It states that the anonymized raw data supporting the findings have been deposited in a public Open Science Framework (OSF) repository, provides the direct URL for access, and describes the contents of the dataset (an Excel file with survey responses from 583 tourists, containing no identifying information). This ensures full transparency and reproducibility.

**Reviewer #2 Comment 7:** 7- Ethical clarity:

Provide more detail on anonymity protection and the information provided to participants.

**Response:** We have expanded the “Ethical Considerations” subsection within the “Data collection” section to provide the requested clarity. The revised text now includes: (1) the name of the approving ethics committee and the approval number; (2) a detailed description of the informed consent procedure, explaining the waiver of written consent due to the anonymous and minimal-risk nature of the survey, and how participants were fully informed via a cover page; and (3) an explicit statement confirming that no personally identifiable information was collected to ensure anonymity.

**Reviewer #2 Comment 8:** 8- Educational experience finding:

Strengthen the explanation for the non-significant direct effect on cultural identity.

**Response:** We have substantially strengthened the explanation for this finding. In the Discussion section, we have added a dedicated subsection titled **“The Indirect Role of Educational Experience in Folk-Religious Contexts.”** Here, we provide an in-depth theoretical explanation, arguing that within the value-laden, symbolic context of folk-religious tourism like Mazu culture, passive knowledge acquisition (educational experience) alone is insufficient to directly foster a deep sense of cultural belonging. Instead, its primary role is to supply the cognitive schemata, which must then align with personal values to spark **emotional resonance.** This affective response, in turn, facilitates the construction of **cultural identity.** This explanation positions the finding not as a null result, but as evidence of a context-specific mechanism where affective alignment precedes cognitive internalization in identity formation.

**Reviewer #2 Comment 9:** Minor suggestions: Standardize “esthetic/aesthetic. Provide descriptive statistics (means, SDs, correlations). Improve Figure 2 by adding coefficients on paths. Avoid strong causal wording due to the cross-sectional design.

**Response:** Standardize “esthetic/aesthetic.”

We have standardized and retained the spelling “esthetic” to strictly adhere to the original terminology of the “4E” framework (Pine & Gilmore, 1999) upon which our experience dimensions are based, ensuring theoretical consistency.

**Response:** Provide descriptive statistics (means, SDs, correlations).

Descriptive statistics (item means and standard deviations) are now provided in S1 Appendix. The correlation matrix for all constructs is presented in Table 4.

**Response:** Improve Figure 2 by adding coefficients on paths.

Figure 2 in the revised manuscript now displays all standardized path coefficients.

**Response:** Avoid strong causal wording due to the cross-sectional design.

We have carefully reviewed the manuscript to avoid overly strong causal language. We use terms such as “influence,” “impact,” and “association” to describe relationships, acknowledging the limitations of causal inference inherent in the cross-sectional design.

**Reviewer #3 Comment 1:** (1)Treatment of Demographic Variables

The current manuscript presents a structural equation modeling (SEM) analysis without addressing whether demographic variables (e.g., gender, education, monthly income, age, occupation) should be incorporated into the model. Considering that demographic characteristics may influence respondents’ perceptions, attitudes, or behavioral outcomes, the authors are encouraged to justify their analytical decision regarding demographic variables. Specifically, if demographic variables were excluded from the SEM model, the manuscript should clearly explain the rationale (e.g., theoretical justification, non-significant preliminary testing, or focus on theoretical constructs rather than population heterogeneity). If demographic variables could potentially influence latent constructs or model outcomes, the authors may consider treating them as control variables, exogenous predictors, or grouping variables in a multi-group SEM framework. Alternatively, if demographic characteristics were analyzed separately (e.g., using t-tests, ANOVA, or subgroup comparisons), such methodological choices should be explicitly described and reported. Clarifying this issue will improve the study’s methodological transparency and strengthen the validity of the structural model’s interpretations.

**Response:** We thank the reviewer for this valuable methodological suggestion. To enhance transparency, we have addressed the treatment of demographic variables in the revised manuscript. Our primary analytical decision was theory-driven: the core aim of this study was to test the universal psychological pathway (the serial mediation model) derived from integrated theories (S-O-R, Resonance Theory, etc.). Therefore, demographic variables were not included as controls in the main SEM to maintain model parsimony and focus on the theoretical constructs of interest.

This decision and its rationale are now explicitly stated in the **“Limitations and Future Research”** section. We acknowledge that while preliminary checks did not indicate strong demographic effects, formally testing demographic heterogeneity remains an important boundary condition. Accordingly, we have incorporated the reviewer's insightful suggestion by proposing that future research explicitly examine demographic variables as moderators or through multi-group SEM, as indicated in the same section. This clarification strengthens the methodological justification of our study.

**Reviewer #3 Comment 2:** (2)Addressing Potential Common Method Variance (CMV)

Because the study appears to rely on self-reported survey data collected from a single source at one point in time, it is susceptible to common method variance (CMV). However, the manuscript currently does not discuss whether CMV was assessed or mitigated. To enhance methodological rigor, the authors should:

Provide a clear statement acknowledging the potential CMV issue inherent in single-source survey-based research.

Describe procedures used to minimize CMV during survey design (e.g., item randomization, anonymity assurances, scale separation, reduced social desirability wording), if applicable.

Include at least one post-hoc statistical diagnostic method to evaluate the extent of CMV. Common approaches include: Harman’s single-factor test; Common latent factor (CLF) approach within the CFA model; Marker variable or unmeasured latent method factor techniques (if appropriate). If CMV was tested, the results and interpretation should be reported. If no CMV assessment was conducted, the manuscript should acknowledge this as a limitation and, where possible, perform supplementary analysis.

Addressing CMV explicitly will increase confidence that observed relationships among latent constructs are substantive rather than artifacts of the measurement method.

**Response:** We have addressed this important concern in the revised manuscript, following the reviewer's recommendations.

**Acknowledgment and Design Measures:** We acknowledge the potential for CMV in the “Reliability and validity testing of scales” section. Procedurally, we ensured respondent anonymity and reduced evaluation apprehension through the survey design and instructions.

**Post-hoc Statistical Diagnostics:** We have implemented and reported the results of two post-hoc statistical tests in the same section: Harman’s single-factor test (single factor explained 36.1% of variance) and a marker variable test (no significant fit improvement, Δχ²(1) = 0.087, p > 0.05). Based on these results, we conclude that common method bias is not a major concern for this study.

These additions provide the necessary methodological transparency and strengthen confidence in the substantive nature of the observed relationships.

**Comment (Academic Editor) 1:** 1. Discriminant validity needs stronger evidence

The manuscript reports Fornell–Larcker values, which is useful, but modern SEM practice requires reporting HTMT ratios (with CIs).

Please add an HTMT matrix to demonstrate the distinctiveness of the constructs, especially emotional resonance, cultural identity, and place identity.“

**Response:** As requested, we have strengthened the evidence for discriminant validity by adding the Heterotrait-Monotrait (HTMT) ratio analysis. The HTMT matrix (Table 5) and the corresponding bootstrap confidence intervals (Table 6) are now reported in the “Reliability and validity testing of scales” section. All HTMT values are well below the conservative threshold of 0.85, confirming the distinctiveness of the constructs, including emotional resonance, cultural identity, and place identity.

**Comment (Academic Editor) 2:** 2. Common Method Bias (CMB) should be assessed more thoroughly

Since all data were collected from a single self-report survey at one time point, CMB is a realistic concern.

The Harman test alone is insufficient. Please apply at least one additional method, such as:

a marker variable,

a latent method factor, or

a common latent factor (CLF) test.

Addressing CMB will improve confidence in the mediation results.

**Response:** We have thoroughly assessed common method bias as advised. In addition to Harman’s single-factor test, we applied a marker variable test as the supplementary method. The results of both tests are now reported in the “Reliability and validity testing of scales” section. The marker variable test showed no significant improvement in model fit (Δχ²(1) = 0.087, p > 0.05), indicating that CMB is not a major concern. This comprehensive assessment strengthens confidence in the mediation results.

**Comment (Academic Editor) 3:** 3. Measurement model details are incomplete

For transparency and reproducibility, please provide:

full item wording (in an appendix),

standardized loadings with SEs,

item means/SDs, and VIF values to rule out multicollinearity.

This information is essential for readers to evaluate construct validity.

**Response:** To ensure full transparency, we have added a comprehensive S1 Appendix that provides: (1) the full wording of all measurement items, (2) their corresponding means and standard deviations (SDs), and (3) the standardized factor loadings with standard errors (SEs) from the confirmatory factor analysis.

Regarding VIF (Variance Inflation Factor) values, they are a diagnostic tool primarily used in regression analysis to assess multicollinearity among observed predictor variables. In our covariance-based structural equation modeling (SEM) framework, the relationships and potential multicollinearity among latent constructs are more conventionally and rigorously assessed through the examination of the construct correlation matrix (Table 4) and the results of the confirmatory factor analysis (Table 3, S1 Appendix), which demonstrate robust discriminant and convergent validity. Therefore, we believe the information now provided offers a complete and standard presentation of the measurement model for evaluation.

**Comment (Academic Editor) 4:** 4. Model fit reporting should include SRMR

The global fit indices are strong, but SRMR is missing.

Please report SRMR and briefly cite the benchmarks used (e.g., Hu & Bentler), to complete the SEM reporting.

**Response:** We have added the SRMR (Standardized Root Mean Square Residual) to all model fit reporting and have cited the recommended benchmarks (Hu & Bentler, 1999) for model fit evaluation in the corresponding text.

**Comment (Academic Editor) 5:** 5. Alternative model comparison is needed

To justify the direction of the sequential mediation, it would be helpful to compare your proposed model against at least one alternative (e.g., identity → resonance, or direct 4E → place identity).

This will strengthen the argument for the proposed causal ordering.

**Response:** We have conducted the recommended alternative model comparison to justify the proposed sequential mediation path. In the “Structural equation modeling testing results” section (and Table 7), we compare our hypothesized model (sequential mediation: 4E → ER → CI → PI) against two theoretically-derived alternative models: one with emotional resonance as the sole mediator (4E → ER → PI) and another with cultural identity as the sole mediator (4E → CI → PI). The results demonstrate the superior explanatory power of our model and statistically validate the necessity of the proposed sequence, thereby strengthening the argument for the specified causal ordering.

**Comment (Academic Editor) 6:** 6. Data availability must meet PLOS requirements

PLOS ONE requires public access to raw data sufficient for replication.

Please deposit:

PLOS ONE

item-level dataset,

analysis code (SPSS/AMOS), and

a short README

in a public repository (e.g., OSF or Zenodo), and update the Data Availability Statement accordingly.

**Response:** We have fully complied with PLOS ONE's data availability policy. As required:

Data and Files Deposited: The anonymized item-level dataset and analysis files have been deposited in a public Open Science Framework (OSF) repository.

Data Availability Statement Updated: A detailed “Data Availability Statement” has been added to the end of the manuscript. It provides the persistent URL to access the repository and describes the contents of the uploaded raw data to ensure reproducibility.

**Comment (Academic Editor) 7:** 7. Ethical procedures need a bit more detail

Please clarify:

whether any identifying information was collected

how anonymity was protected, and

what participants were told before consenting。

These additions will strengthen the ethics section.

**Response:** We have expanded the “Ethical Considerations” subsection within the “Data collection” section to provide all requested details:

Identifying Information: We explicitly state that no personally identifiable information was collected.

Anonymity Protection: Anonymity was ensured through the aforementioned non-collection of personal data and the completely anonymous administration of the survey.

Informed Consent Process: We detail that participants were presented with a cover page outlining the research purpose, procedures, data usage, voluntary nature, confidentiality, and right to withdraw. Proceeding with the questionnaire constituted implied consent. This procedure was approved by the Putian university ethics committee (Approval No.: Ethical Review (2025)020).

**Comment (Academic Editor) 8:** 8. Interpretation of the non-significant Educational → Cultural Identity path

This is an interesting and important finding, but the current explanation is brief.

Please elaborate on why educational experiences did not directly influence cultural identity in this context and relate this to prior literature on folk-religious tourism or experiential learning.

**Response:** We have substantially elaborated on this key finding in the revised manuscript. In the **Discussion** section, a dedicated subsection titled **“The Indirect Role of Educational Experience in Folk-Religious Contexts”** provides an in-depth explanation.

We argue that within the **value-laden, symbolic context of folk-religious tourism** (exemplified by Mazu culture), passive knowledge acquisition alone is insufficient to directly forge a deep, internalized sense of cultural belonging (identity). Instead, drawing on **Resonance Theory** and **Fredrickson’s Broaden-and-Build Theory,** we posit that the primary role of educational experience is to provide the necessary **cognitive schemata** (narratives, values). For this knowledge to translate into identity, it must first align with the tourist’s personal values, thereby triggering **emotional resonance.** This profound affective response then broadens cognitive resources and facilitates the construction of **cultural identity.**

This explanation moves beyond a mere statistical observation to propose a **context-specific theoretical mechanism:** in folk-religious tourism where identity is deeply tied to symbolic attachment, the path from cognitive learning to identity is **effectively gated by affective evaluation.** This elaboration directly relates our finding to the distinct nature of experiential learning in heritage and religious settings, addressing the editor’s request.

**Comment (Academic Editor) 9:** B- Minor Suggestions

Standardize the terminology (“esthetic” vs. “aesthetic”).

Provide descriptive statistics (means, SDs, correlations) for all variables.

Improve figure clarity by adding coefficients directly on paths.

Avoid strong causal wording because the design is cross-sectional.

A brief proofread would smooth minor language issues.

**Response:** We have addressed all minor suggestions as follows:

**Terminology:** We standardized and retained **“esthetic”** throughout to adhere strictly to the original “4E” framework terminology.

**Descriptive Statistics:** Item-level means and standard deviations are provided in **S1 Appendix,** and construct-level correlations are reported in **Table 4.**

**Figure Clarity: Figure 2** has been updated to display all standardized path coefficients directly on the paths.

**Causal Wording:** We have carefully reviewed the manuscript to avoid strong causal claims, using terms like “influence” and “association” appropriate for the cross-sectional design.

**Proofreading:** The manuscript has undergone thorough proofreading to correct minor language issues and improve overall fluency.

Once again, we thank the editors and reviewers for their valuable guidance. We believe the manuscript has been substantially improved through this revision process and hope it now meets the high standards of PLOS ONE.

Sincerely,

Wen Qiang Chen, on behalf of all co-authors

Putian University

wenqiang1990@hotmail.com
