# Supplementary material for: The impact of cultural tourism experience on cultural identity: A case study of Mazu culture
Source: PLoS One. 2026 Apr 2;21(4):e0346153. doi: 10.1371/journal.pone.0346153 (PMC13046174; doi:10.1371/journal.pone.0346153)
Supplement: Attachment — Submitted filename: Response_to_Reviewers_auresp_2.docx [file pone.0346153.s006.docx]

Response to Reviewers

Manuscript ID PONE-D-25-53833

Title: The Impact of Cultural Tourism Experience on Cultural Identity: A Case Study of Mazu Culture

Dear Dr. Zhu and Reviewers,

Thank you for the opportunity to revise our manuscript. We sincerely appreciate your time and the constructive feedback provided, which has been invaluable in strengthening our paper. We have carefully addressed all comments, and the changes are highlighted in the submitted ‘Revised Manuscript with Track Changes’ file.

Below, we provide a point-by-point response.

**Response to Comments from Reviewer #1:**

**Reviewer #1 Comment 1:** Literature review

The conceptual model should be at the end of the literature review not beginning.

**Response:** As recommended, we have relocated the entire “Theoretical Research Model” subsection (including Fig 1) to the end of the “Literature review and hypotheses development” section. This adjustment provides a clearer synthesis of the integrated model after all theoretical foundations and hypotheses (H1–H19) have been presented, improving the logical flow before the Methods section. The subsection is now placed at the end of the “Literature review and hypotheses development” section (see Page 13-14).

**Reviewer #1 Comment 2:** The literature review lacks critical analysis or discussion of alternative models or critiques to the 4 realms of the experience economy. It reads more like a summary than a critical synthesis. To strengthen this section, I recommend incorporating more targeted literature on the 4Es applied to cultural tourism experiences, including critical perspectives and empirical findings that reflect cultural specificity and complexity.

**Response:** We have fundamentally revised the ‘Literature review and hypotheses development’ section to move beyond summary and provide a critical synthesis of the 4E framework within our specific research context. The revisions are detailed below:

**Explicit Critique of the 4E Framework’s Contextual Limits:** We now explicitly problematize the direct application of the 4E model—derived predominantly from Western or secular settings—to a value-laden, folk-religious context like Mazu culture. A dedicated paragraph in the ‘Cultural tourism experience’ subsection (Page 6-7) argues that the nature and salience of the 4E dimensions may be reconstituted in such an environment. For instance, we critically posit that the ‘Escapist’ experience here likely encompasses a deeper quest for spiritual solace rather than mere recreational diversion, and the ‘Educational’ dimension may function more as engagement with identity-reinforcing narratives than passive knowledge acquisition.

**Incorporation of Targeted Literature and Critical Perspective:** Guided by the reviewer’s recommendation, we have integrated targeted discussions that anchor the 4E framework within cultural tourism scholarship while highlighting its complexities:

We cite and engage with studies that have applied the 4E framework in cultural tourism (e.g., Juliana et al., 2024; Oh et al., 2007), while simultaneously noting the persistent gap in understanding its “unique dimensions and linkages to psychological outcomes” in such settings (Page 7).

We directly link the need for this critical examination to identified research gaps, stating that the framework’s application in culturally specific settings is “limited” and that related theories are “rarely validated in religious cultural contexts” (Page 7). This creates a critical through-line from the identified gap to our analytical approach.

**Critical Synthesis Through Contextual Re-interpretation:** The entire hypothesis development process now serves as a critical synthesis. We do not merely list the 4E dimensions; we theorize how each might operate differently within Mazu culture and why it matters for our chain of outcomes. For example:

The hypothesis for ‘Escapist → Emotional Resonance’ (H3) is grounded in its role in fulfilling needs for “spiritual transcendence” (Page 7), directly reflecting the cultural complexity noted by the reviewer.

The discussion of the non-significant direct path from ‘Educational experience’ to ‘Cultural Identity’ (Page 32, ‘The Indirect Role of Educational Experience’) in the Results and Discussion sections provides an empirical critique and nuanced understanding of how this 4E dimension functions in our specific folk-religious context, affirming that its role is more complex than a generic model would suggest.

In summary, we have transformed the narrative from a descriptive summary to a critical evaluation that: (a) questions the universal application of the 4E model, (b) contextualizes its dimensions within the cultural specificity of folk-religious tourism, and (c) uses our empirical findings to reflect on its complex mechanisms. We believe these revisions have substantially strengthened the theoretical grounding and analytical depth of our literature review.

**Reviewer #1 Comment 3:** Methodology

Data analysis section is missing. Please explain and justify SEM. PLS-SEM!

Software used.

Type of sampling.

**Response:** We have added a detailed “Data Analysis” subsection (Page 17) to address these points transparently:

SEM Justification: We employed covariance-based SEM (CB-SEM) in AMOS 24.0. Our primary aim is theory testing of a well-established *a priori* model, for which CB-SEM is the standard approach for confirmatory analysis with reflective measures. The sample size (N=583) is sufficient for this method.

Software: Data preparation used SPSS 26.0; CFA and SEM were conducted with AMOS 24.0.

Sampling: We used a convenience sampling approach for pragmatic data collection at the field site. We acknowledge this as a limitation for generalizability, which is discussed in the “Limitations” section (Page 18).

**Reviewer #1 Comment 4:** Results. There seems not be any reference in the text about table 4.

**Response:** We have now explicitly referenced and discussed Table 4 in the main text. The reference is located in the “Reliability and validity testing of scales” subsection (Page 22). The added text reads: *“Discriminant validity was assessed... As shown in Table 4, the square root of each construct’s AVE (diagonal) exceeded its correlations with other constructs (off-diagonal), satisfying the Fornell-Larcker criterion.”*

**Reviewer #1 Comment 5:** Discussion and conclusion are too short.
In conclusion you should answer to your aims and provide clear stance of your work.
Consider moving theoretical and practical implications to conclusion section (not mandatory).

**Response:** We have substantially revised and expanded both the Discussion and Conclusion to address these points directly.

The Discussion now provides deeper interpretation of key findings (e.g., centrality of escapist experience, Page 31-34).

The Conclusion has been rewritten to directly recap and answer the three research aims stated in the introduction, providing a clear final stance on the validated pathway (Page 37).

Regarding implications, we maintained their dedicated sections (‘Theoretical’ and ‘Practical Implications’) to preserve a logical narrative flow, as suggested by the reviewer’s note that this change was not mandatory. The essence of these implications is effectively synthesized within the concluding paragraph.

Once again, we thank the editors and reviewers for their valuable guidance. We believe the manuscript has been substantially improved through this revision process and hope it now meets the high standards of PLOS ONE.

Sincerely,

Wen Qiang Chen, on behalf of all co-authors

Putian University

wenqiang1990@hotmail.com
