# Supplementary material for: The impact of cultural tourism experience on cultural identity: A case study of Mazu culture
Source: PLoS One. 2026 Apr 2;21(4):e0346153. doi: 10.1371/journal.pone.0346153 (PMC13046174; doi:10.1371/journal.pone.0346153)
Supplement: Attachment — Submitted filename: Response_to_Reviewers_auresp_3.docx [file pone.0346153.s007.docx]

**Response to Reviewers**

Manuscript ID PONE-D-25-53833

Title: The Impact of Cultural Tourism Experience on Cultural Identity: A Case Study of Mazu Culture

Dear Dr. Zhu and Reviewers,

Thank you for the opportunity to further revise our manuscript. We have carefully addressed all remaining comments, and the changes are highlighted in the submitted ‘Revised Manuscript with Track Changes’ file.

Below is our point-by-point response.

**Response to the Academic Editor:**

**Comment:** A comprehensive comparison of your overall results with existing studies is important.

**Response:** We agree and have substantially expanded the Discussion to provide a systematic comparison of our core findings with prior literature. Each major finding (escapist experience, educational experience, emotional resonance, direct effects of entertainment/esthetic experiences, and cultural identity→place identity) is now explicitly contrasted with relevant studies, with explanations for consistencies and divergences. These revisions appear on Pages 31–34.

**Response to Reviewer #1:**

**Comment 1:** In the discussion section, you need to more clearly contrast your findings with the previous literature. At present, a comprehensive comparison of your overall results with existing studies is missing.

**Response:** As detailed above, we have thoroughly revised the Discussion to include a comprehensive comparison with existing studies. Key contrasts include:

Escapist experience: compared with Juliana et al. (2024), Allan (2016), Liu and Lin (2024)

Educational experience: contrasted with Urošević (2012), supported by Zhang and Liu (2025)

Emotional resonance: compared with Qu et al. (2025), Yang et al. (2023), Hosany et al. (2016), Wang et al. (2023)

Entertainment/esthetic experiences: compared with Urošević (2012), Juliana et al. (2024), Allan (2016)

Cultural identity→place identity: compared with Casais and Poço (2023), Wang et al. (2023), Cao et al. (2021)

Please see Pages 31–34.

Comment 2: The Limitations and Future Research section should be placed at the end of the conclusion, immediately before the references.

Response: We have moved the “Limitations and Future Research” section to the end of the Conclusion, immediately before the References.

Please see Page 39.

Comment 3: Given the number of subheadings in the discussion, it would improve readability if the theoretical and practical implications were clearly numbered.

Response: We thank the reviewer for this suggestion. After checking the journal’s formatting guidelines, we found that numbered subheadings are not permitted in the manuscript. To maintain compliance with the journal’s style requirements, we have retained the standard unnumbered headings. We have, however, ensured that the subheadings are clearly formatted (bolded) to enhance readability.

Comment 4: Also, please note that you wrote "reference" instead of "references."

Response: We have corrected this throughout the manuscript. The heading is now “References” consistently.

We thank the editors and reviewers for their valuable guidance. We believe the manuscript has been substantially improved and hope it now meets the standards of PLOS ONE.

Sincerely,

Wen Qiang Chen, on behalf of all co-authors

Putian University

wenqiang1990@hotmail.com
