# Supplementary material for: The impact of cultural tourism experience on cultural identity: A case study of Mazu culture
Source: PLoS One. 2026 Apr 2;21(4):e0346153. doi: 10.1371/journal.pone.0346153 (PMC13046174; doi:10.1371/journal.pone.0346153)
Supplement: Attachment — Submitted filename: Reviewer Comments for Manuscript PONE-D-25-53833.docx [file pone.0346153.s003.docx]

1- **Reviewer Comments to the Authors**

**Manuscript ID:** PONE-D-25-53833
**Title:** *The Impact of Cultural Tourism Experience on Cultural Identity: A Case Study of Mazu Culture*

**Recommendation:** Major Revision

The manuscript explores how different dimensions of cultural tourism experience (Entertainment, Educational, Escapist, Esthetic) influence emotional resonance, cultural identity, and place identity in the context of Mazu culture on Meizhou Island. The topic is meaningful and the writing is generally clear, and the authors present a well-motivated theoretical framework supported by a large dataset and structural equation modeling.

However, despite these strengths, the manuscript requires several important methodological and reporting improvements before it can meet PLOS ONE’s standards for technical rigor and transparency.

**A- Major Issues (Require Attention Before Acceptance)**

**1. Discriminant validity needs stronger evidence**

The manuscript reports Fornell–Larcker values, which is useful, but modern SEM practice requires reporting **HTMT ratios** (with CIs).
Please add an HTMT matrix to demonstrate the distinctiveness of the constructs, especially emotional resonance, cultural identity, and place identity.

**2. Common Method Bias (CMB) should be assessed more thoroughly**

Since all data were collected from a single self-report survey at one time point, CMB is a realistic concern.
The Harman test alone is insufficient. Please apply at least one additional method, such as:

- a marker variable,
- a latent method factor, or
- a common latent factor (CLF) test.

Addressing CMB will improve confidence in the mediation results.

**3. Measurement model details are incomplete**

For transparency and reproducibility, please provide:

- full item wording (in an appendix),
- standardized loadings with SEs,
- item means/SDs, and
- VIF values to rule out multicollinearity.

This information is essential for readers to evaluate construct validity.

**4. Model fit reporting should include SRMR**

The global fit indices are strong, but SRMR is missing.
Please report SRMR and briefly cite the benchmarks used (e.g., Hu & Bentler), to complete the SEM reporting.

**5. Alternative model comparison is needed**

To justify the direction of the sequential mediation, it would be helpful to compare your proposed model against at least one alternative (e.g., identity → resonance, or direct 4E → place identity).
This will strengthen the argument for the proposed causal ordering.

**6. Data availability must meet PLOS requirements**

PLOS ONE requires public access to raw data sufficient for replication.
Please deposit:

- item-level dataset,
- analysis code (SPSS/AMOS), and
- a short README

in a public repository (e.g., OSF or Zenodo), and update the Data Availability Statement accordingly.

**7. Ethical procedures need a bit more detail**

Please clarify:

- whether any identifying information was collected,
- how anonymity was protected, and
- what participants were told before consenting.

These additions will strengthen the ethics section.

**8. Interpretation of the non-significant Educational → Cultural Identity path**

This is an interesting and important finding, but the current explanation is brief.
Please elaborate on why educational experiences did not directly influence cultural identity in this context and relate this to prior literature on folk-religious tourism or experiential learning.

**B- Minor Suggestions**

- Standardize the terminology (“esthetic” vs. “aesthetic”).
- Provide descriptive statistics (means, SDs, correlations) for all variables.
- Improve figure clarity by adding coefficients directly on paths.
- Avoid strong causal wording because the design is cross-sectional.
- A brief proofread would smooth minor language issues.

**C- Strengths of the Paper**

- Well-defined conceptual framework integrating several relevant theories.
- Strong motivation grounded in UNESCO intangible cultural heritage.
- Large sample size (N=583) and good model fit.
- Important implications for cultural tourism management.

These strengths position the manuscript well, provided the methodological issues above are addressed.

2- **Confidential Comments to the Editor**

This is a valuable and relevant study, but essential methodological clarifications—especially regarding discriminant validity, CMB remedies, data availability, and model comparison—are needed before the manuscript can be considered technically sound under PLOS ONE criteria.
